# Case Report: Filamin C gene mutation associated with restrictive cardiomyopathy leading to heart transplantation

**DOI:** 10.3389/frtra.2024.1431851

**Published:** 2024-12-23

**Authors:** Ludmila De Oliveira Jaime Sales, Paulo Sampaio Gutierrez, Adailson Wagner D. Siqueira, Marcelo Biscegli Jatene, Estela Azeka

**Affiliations:** Pediatric Cardiology and Adult with Congenital Heart Disease Unit, Instituto do Coração (InCor) do Hospital das Clinicas da Faculdade de Medicina da Universidade de São Paulo, São Paulo, Brazil

**Keywords:** restrictive cardiomyopathy, desmin, Filamin C, heart transplantation, heart failure

## Abstract

**Background:**

Cardiomyopathy is a disease that affects the myocardium and can be classified as dilated, restrictive, or hypertrophic cardiomyopathy. Among the subtypes, restrictive cardiomyopathy is characterized by restriction of ventricular filling and its uncommon cause is a disease due to mutation on Filamin C (FLNC) gene. Filamin C is an actin-binding protein encoded by FLNC gene and participates in sarcomere stability maintenance, which is expressed on the striated muscle. FLNC variants have been associated with restrictive cardiomyopathy and non-compaction cardiomyopathies. The association of FLNC with a broad spectrum of cardiac phenotypes shows an important gap in knowledge. Therefore, a wide investigation is necessary to diagnose this pathology, including an anatomopathological study and genetic tests.

**Methods/results:**

The purpose of this study is to report about a patient who had restrictive cardiomyopathy due to mutation on Filamin C gene and was indicated for heart transplantation.

**Conclusion:**

The etiology of cardiomyopathy is important for the clinical management of the patient and also for guiding families regarding genetic counseling and prevention of new cases in the family.

## Introduction

Cardiomyopathies are defined by primary involvement of the myocardium, and can be characterized as hypertrophic, dilated, and restrictive. Restrictive cardiomyopathy is characterized by a rigid, non-dilated ventricle with severe diastolic dysfunction and restricted ventricular filling that progressively leads to symptoms of heart failure and diseases of the heart's electrical conduction system (1, 2). Several etiologies have been described, such as infiltrative, inflammatory, toxic, genetic, and idiopathic disease (3). Among the genetic causes, the mutation in the Filamin C (FLNC) gene stands out. Filamin C is an actin-binding protein and participates in sarcomere stability maintenance of the striated muscles.

FLNC was first demonstrated to be among the causal gene of myofibrillar myopathies (MFM), a group of myopathies that have common pathological changes, such as cytoplasmic hyaline inclusions with various sizes and shapes, best observed in trichrome-stained sections (4). In anatomopathological studies, deposits of desmin and some sets of myofibrillar and non-myofibrillar proteins can be found in or around these inclusions, at both muscle and cardiac levels, causing disarray in the cellular cytoskeleton and compromising cellular integrity (4–6).

MFM mutations have been identified in genes encoding desmin, αB-crystallin, myotilin, Z-band alternatively spliced PDZ motif protein, Filamin C, Bag3, and FHL1. However, it has been found that FLNC mutation plays a critical role in the pathogenesis of cardiomyopathy (1, 7).

The etiological definition of cardiomyopathy due to FLNC mutation usually requires a wide propaedeutic, including anatomopathological study and genetic tests (8). This article aims to report and discuss a case of a patient who underwent heart transplantation due to cardiac dysfunction and the accumulation of desmin in the myocardium, which was evidenced in the anatomopathological examination, and FLNC mutation, which was found in genetic test.

## Methods

This study reports about a patient with restrictive cardiomyopathy due to FLNC mutation, which complicated clinical management and lead to the indication for heart transplantation. This study was conducted through a review of the electronic medical record (Si3) and a literature review.

### Case report

A 5-year-old female child presented with dyspnea and recurrent pulmonary infections. There were no significant pathological antecedents in the prenatal or perinatal periods. Following initial investigations, when she was 7 years old, she was diagnosed with restrictive cardiomyopathy and started on medications for heart failure. In addition to cardiovascular symptoms, the patient presented with non-specific weakness and elevated serum creatine phosphokinase (CPK) levels, which led to the investigation of some type of myopathy. There was no history of muscle strength loss, gait disturbances, or movement restrictions.

At the age of 14, the patient was referred for evaluation for heart transplantation due to worsening heart failure symptoms, which required frequent hospitalizations for clinical management and deterioration of functional class despite optimized doses of anti-congestive medications. At the time of admission, the patient was using aspirin 100 mg once a day, enalapril 5 mg every 12 h, carvedilol 3.125 mg every 12 h, furosemide 40 mg every 12 h, spironolactone 25 mg once a day, and hydrochlorothiazide 25 mg daily. Complementary exams conducted on admission included transthoracic two-dimensional echocardiography, which demonstrated significant dilation of the right and left atria, with ventricles of normal dimensions and function. Cardiac magnetic resonance imaging confirmed left atrial enlargement (left atrial volume index: 157 ml/m^2^), with no signs of myocardial edema. Marked hypertrabeculation was observed in both ventricles, especially in the apical and lateral segments, with a non-compacted/compacted left ventricle (LV) myocardium ratio estimated at 3.3, in addition to malformed papillary muscles. These findings confirmed the diagnosis of restrictive cardiomyopathy. Cardiac catheterization revealed an increase in pulmonary vascular resistance (PVR: 5.5 WU/m^2^), mean pulmonary pressure of 23 mmHg, and wedge pressure of 18 mmHg.

The patient was receiving triple therapy for the treatment of heart failure with aspirin, ACE inhibitors, diuretics, and beta-blockers, and despite preserved ventricular function on imaging studies, the patient exhibited frequent symptoms of heart failure, such as dyspnea with minimal exertion, nausea and vomiting, and abdominal pain, even after optimizing the medication treatment. The patient was subsequently listed for heart transplantation and required continuous vasoactive drugs to improve heart failure symptoms before undergoing the procedure.

**Figure 1:**
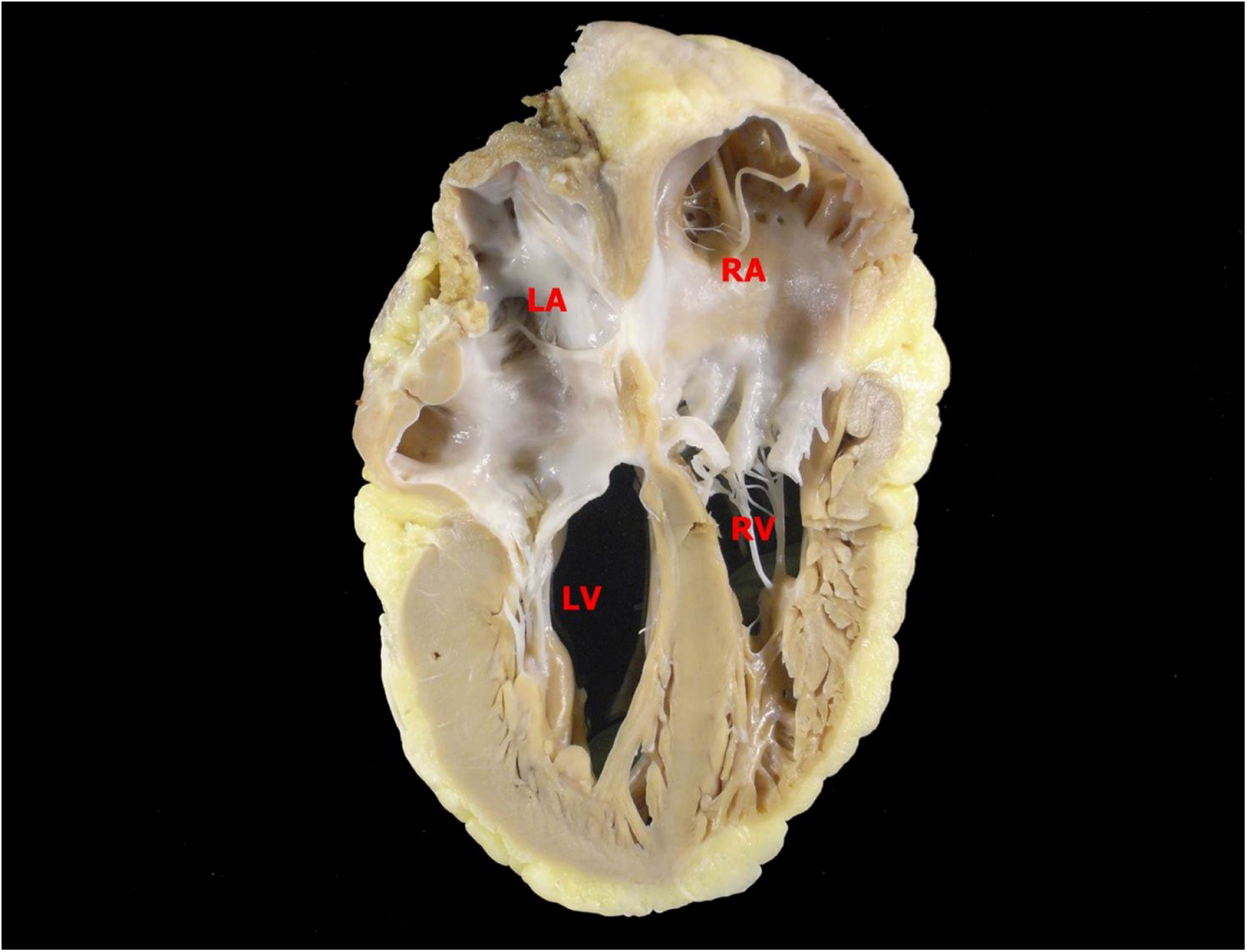
Posterior view of a longitudinal section of the heart. Atria and ventricles are similar in height, an aspect that resembles a restrictive pattern of cardiomyopathy. LA, left atrium; RA, right atrium; LV, left ventricle; RV, right ventricle.

**Figure 2:**
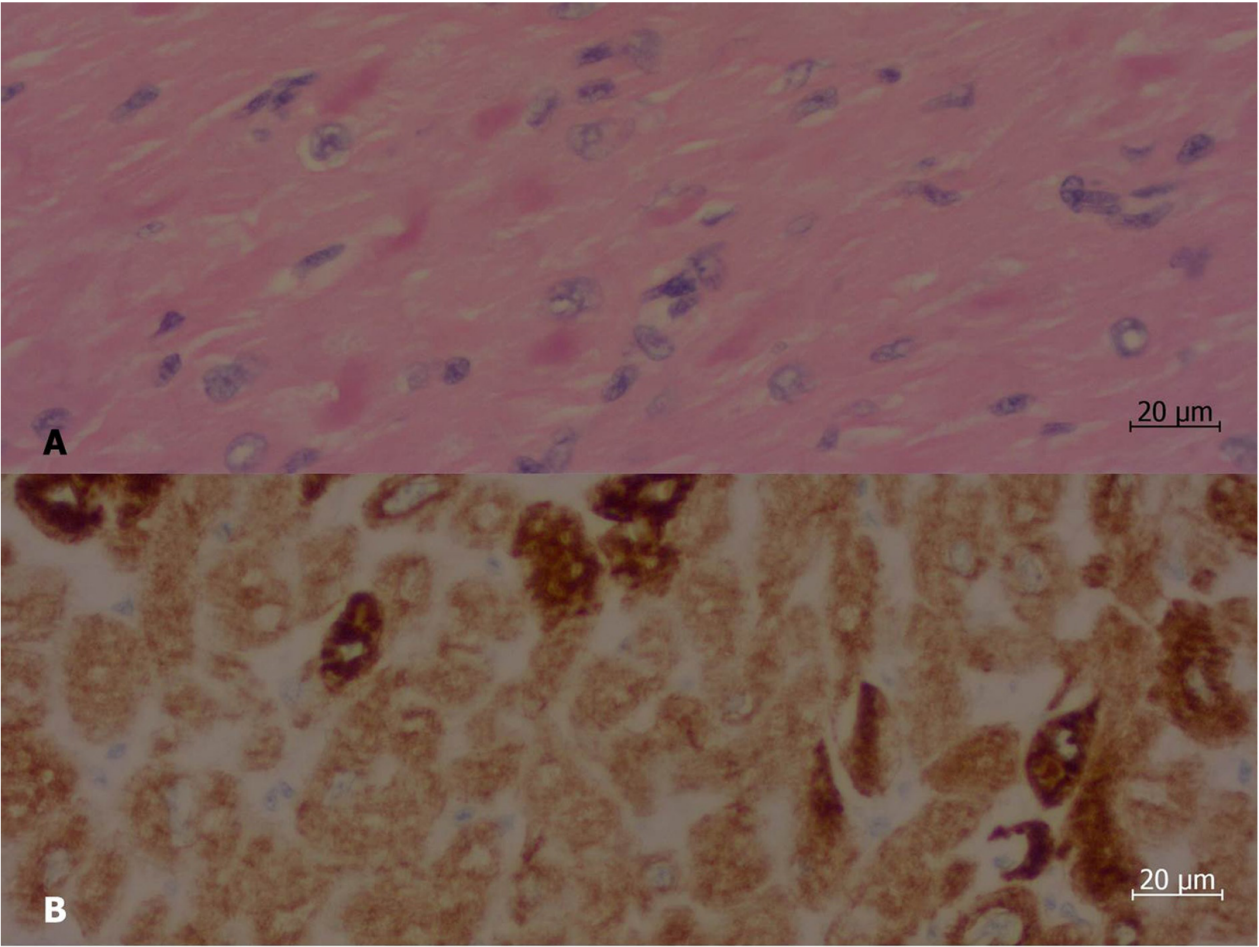
Histological section of a sample of the left ventricle of the explanted heart. **(A)** At hematoxylin and eosin staining, there were irregular, strongly eosinophilic deposits (stained in deep pink) inside some of the myocardiocytes. **(B)** Immunohistochemical reaction demonstrated that such deposits are positive for desmin (stained in deep brown). Objective magnification: 40×.

The transplant occurred without complications after less than a year on the list. At the anatomopathological examination, the heart weighed 310 g, with enlarged atria contrasting with the ventricles. On cross-sections, slight dilation of the ventricular cavities was noted. The myocardium exhibited trabeculations within normal limits. Representative samples submitted for microscopic examination showed cardiomyopathy with a restrictive pattern (disproportionate atrial dilation and hypertrophy relative to the ventricles—Figure 1), hypertrophy of cardiomyocytes with focal presence of eosinophilic sarcoplasmic deposits, positive for desmin filaments (immunohistochemical reaction—Figure 2), which further confirmed the diagnosis.

Further investigation revealed a genetic mutation in the *Filamin C* gene (heterozygous variant *c.5483C>T*), the variant found promotes the substitution of the amino acid proline at codon 1828 with leucine (*p.Pro1828Leu*), which was considered a variant of uncertain significance. It is to be noted that the patient's father also had cardiomyopathy and passed away while on the transplant list before the patient received her diagnosis. He was not subjected to any genetic testing at the time of his follow-up. It was therefore considered that the father would likely be a carrier of a similar mutation, and no investigation was conducted on other family members, since the mother was asymptomatic and the patient in question had no siblings.

A muscle biopsy showed increased lipid deposits with no desmin deposits visualized, and electromyoneurography showed no alterations.

Currently, the patient is undergoing regular specialized follow-up to manage the heart transplant and prevent complications. She is appropriately using immunosuppressants, with good laboratory and clinical control after transplantation. Regarding skeletal muscle symptoms, the patient remains stable, maintaining elevated blood levels of muscle enzymes (CPK) but without limitations in daily activities or progression of proximal muscle weakness.

## Discussion

This case identified a mutation in the *FLNC* gene. Similar to other filamins, FLNC is a structural protein whose main role is to maintain the structural integrity of the sarcomere (9). Unlike Filamin A and B, *FLNC* expression is restricted to striated muscles and is localized around the Z-disc, the sarcolemma, the myotendinous junction, and the intercalated discs.

The proper functioning of *FLNC* is crucial for maintaining structural integrity and cell signaling within the sarcomere, thus ensuring the adequate functioning of striated muscle cells, including those in cardiac tissue. Over 200 variants of the *FLNC* gene have been described in the literature, most of which are recognized as disease-associated genes in the context of cardiomyopathies (9, 10). While mutations are more commonly associated with dilated cardiomyopathies, the literature also documents cases linked to restrictive cardiomyopathies and other forms of cardiac diseases.

Genetic testing is essential for accurately diagnosing this disease, which is often underdiagnosed—a fact confirmed by the presented case, as the condition was only diagnosed after several years of monitoring. *FLNC* gene mutations are associated with muscular conditions, sudden death, and arrhythmogenic heart disease. While the association with the dilated cardiomyopathy phenotype is more prevalent, the literature indicates an association with restrictive cardiomyopathy in 8% of cases (7, 11).

Muscle involvement may present as proximal or distal muscle weakness, depending on the mutation variant. The condition may evolve progressively, leading to the need for mechanical ventilation and an inability to walk, or it may not manifest in the clinical picture. Nevertheless, regardless of genetic variation, persistent high levels of creatine kinase (CK) are commonly observed, as noted in this case, where levels can be elevated up to tenfold above the upper limit (1).

Electromyography typically reveals myopathic changes; however, in this case, the patient exhibited a normal electromyoneurographic study with no evidence of myopathy, and a muscle biopsy indicated only lipid deposits without relevant peripheral nervous system involvement. This finding contrasts with the literature indicating neurogenic changes in approximately half of skeletal muscle biopsies from patients with *FLNC* mutations (1, 12).

This case documents a female patient with a typical presentation of skeletal myopathy and restrictive heart disease that progressed to heart failure, necessitating a transplant. While there is no specific treatment for *FLNC* mutations, complications and early mortality can be mitigated through proper diagnosis and specialized follow-up. As demonstrated in this report, the patient experienced a progressive course of heart disease despite adequate and optimized clinical management, ultimately leading to heart transplantation. However, she continues to experience skeletal muscle symptoms, albeit without progression at this time.

This study illustrates the clinical evolution of restrictive cardiomyopathy due to *FLNC* mutations, which leads to progressive diastolic dysfunction and the need for heart transplantation. As this is a genetic condition, most affected patients and their families require regular follow-up to identify complications and ensure early diagnosis. In this case, since the patient was considered the only living carrier of the mutation, no genetic investigation was conducted on other family members. The patient received guidance regarding genetic counseling.

## Conclusion

Cardiomyopathies exhibit numerous forms of presentation and potential etiologies, making it vital to understand them to properly investigate, diagnose, and treat affected patients. The *FLNC* gene mutation is one of the mutations increasingly studied in association with cardiomyopathy. This case presents a patient with an *FLNC* gene mutation who developed restrictive cardiomyopathy leading to ventricular dysfunction and, consequently, heart transplantation. Therefore, genetic counseling for the affected patient is critical to identify gene mutations and provide appropriate guidance for the affected family.

## Data Availability

The raw data supporting the conclusions of this article will be made available by the authors upon request, without undue reservation.
